# A metal–organic framework neuron

**DOI:** 10.1093/nsr/nwaf213

**Published:** 2025-05-23

**Authors:** Zheng Li, Miao-Hua Chen, Qing-Qing Wu, Cheng Yuan, Jing-Juan Xu, Hong-Yuan Chen, Wei-Wei Zhao

**Affiliations:** State Key Laboratory of Analytical Chemistry for Life Science, School of Chemistry and Chemical Engineering, Nanjing University, Nanjing 210023, China; State Key Laboratory of Analytical Chemistry for Life Science, School of Chemistry and Chemical Engineering, Nanjing University, Nanjing 210023, China; State Key Laboratory of Analytical Chemistry for Life Science, School of Chemistry and Chemical Engineering, Nanjing University, Nanjing 210023, China; State Key Laboratory of Analytical Chemistry for Life Science, School of Chemistry and Chemical Engineering, Nanjing University, Nanjing 210023, China; State Key Laboratory of Analytical Chemistry for Life Science, School of Chemistry and Chemical Engineering, Nanjing University, Nanjing 210023, China; State Key Laboratory of Analytical Chemistry for Life Science, School of Chemistry and Chemical Engineering, Nanjing University, Nanjing 210023, China; State Key Laboratory of Analytical Chemistry for Life Science, School of Chemistry and Chemical Engineering, Nanjing University, Nanjing 210023, China

**Keywords:** neuromorphic, metal–organic framework, neuron, spike, real neurotransmitter

## Abstract

The demand for deep human–machine fusion propels the development of artificial neurons. However, emulating the neuronal spiking in aqueous environments remains challenging. Metal–organic frameworks (MOFs) have recently shown promise in neuromorphic engineering compatible with aqueous operation. Here, we report a MOF neuron with real neurotransmitter—dopamine (DA)—tunable spikes for the first time. Based on the DA mediation, some sophisticated neuronal functions, including integration-and-firing, synaptic facilitation-induced spike broadening and DA-tunable spiking number and width, were mimicked. DA-mediated spikes in this MOF neuron were further implemented to exquisitely control peripheral equipment. This work introduces the concept of a MOF neuron interfaced with a real neurotransmitter in fluids, providing a new perspective for artificial neuron development.

## Teaser

This study presents the first metal-organic framework neuron with real neurotransmitter dopamine tunable spikes for neuromorphic engineering.

## INTRODUCTION

The intricate operation of the human brain has inspired the development of various innovative neuromorphic devices towards e.g. artificial afferent nerves, antennal sensory systems and neuromuscular systems [1–4]. Neurons are fundamental units of the human nervous system that process and transmit information by using chemical synapses via spikes or action potentials (APs) [5,6]. The generation and properties of the spikes are collectively influenced by voltages, ions and neurotransmitters in a liquid medium [7,8]. To encode the rich information, neurons exhibit diverse firing behaviors, as indicated by the spikes with a wide range of shapes, frequencies and patterns [5]. Thus, to create realistic analogs to the neurons, one should emulate the chemically synaptic transmission [9], which involves ions [10] and neurotransmitters [11–13], and the changeable spike properties [14], which alters in e.g. width and height. Namely, ideal artificial biosensory neurons should simultaneously perform characteristic spiking and biological perception. Existing solid-state neurons, based on silicon or metal-oxide semiconductors [15–18], fail to work in aqueous environments, restraining their neurotransmitter/ion-modulated neural features. More realistic aqueously compatible neurons can be achieved by using organic neurons based on mixed ion–electron conducting polymers [19–21]. However, the challenges for the development of organic neurons are concerns about structural and performance requirements, making the laborious design and synthesis of functional polymers a bottleneck to new discoveries [22] (Table S1).

Metal–organic frameworks (MOFs) constitute a class of porous crystalline materials assembled from metal ionic centers and organic molecular linkers through strong bonds [23,24]. Due to their unique properties, e.g. structural designability, tunability and controllability, MOFs have found applications in diverse sectors [25–28]. Especially, attracted by its rich chemistry, high porosity, volumetric capacitance, unique memristive properties, etc. [29–31], MOFs have recently gained attention in the field of aqueous neuromorphic devices [32–36]. For example, Kim *et al.* constructed an artificial retina system in which P3HT served as the channel while the MOF layer was an ion-limiting interface [32]. Tang *et al.* reported a supercapacitor–memristor through non-linear ion transport in MOF nanochannels for neuromorphic emulation [33]. Wang *et al.* used a photosensitive MOF membrane in a 3-terminal nanofluidic transistor to optically implement synaptic functions [34]. Our group earlier employed MOFs in two-terminal nanofluidic memristors for artificial synapses [36]. Despite the progress, the research in this field is still young.

**Figure 1.**
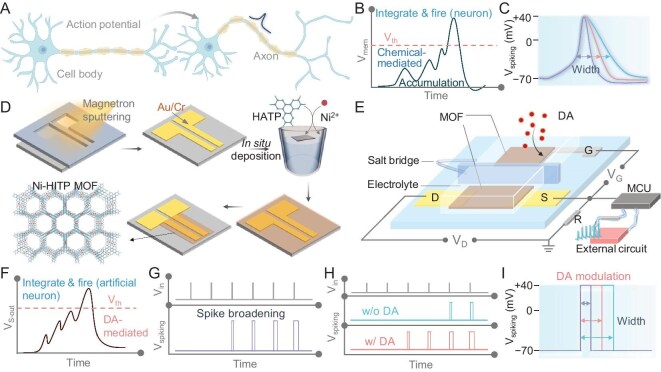
MOF neuron. (A) Schematic of the biological signal transmission mediated by spikes. (B) Schematic of the integrate-and-fire (I&F) model of biological neurons. (C) Schematic of the biological spikes with different widths. (D) Schematic of the fabrication process of the MOF transistor. (E) Schematic of the MOF neuron consisting of a MOF transistor with a salt bridge-equipped H-cell, an MCU and an external circuit. The resistor (*R*) value was equal to the initial resistance of the device. Schematic of the (F) I&F model, (G) spike broadening, (H) DA-mediated spike number and (I) DA-mediated spike width.

In this article, we report the first three-terminal MOF neuron with real neurotransmitter—dopamine (DA)—tunable spiking in an aqueous environment. In biological neurons (Fig. 1A), signaling transmission is mediated by spikes that comply with the neurotransmitter-mediated integrate-and-fire (I&F) model (Fig. 1B), in which the spike shapes, including the width, are used to encode information (Fig. 1C) [5]. Inspired by this, the MOF neuron with DA perception was developed. Specifically, a semiconductive MOF [37], Ni_3_(HITP)_2_ (HITP = 2,3,6,7,10,11-hexaiminotriphenylenesemiquinonate) [38], was *in situ* deposited onto the patterned substrate to prepare a MOF transistor (Fig. 1D and Fig. S1) [31,39]. The materials properties and transistor characteristics were then investigated (Figs S2–S9). Such a MOF transistor was further linked with a microcontroller unit (MCU) and an external circuit to construct the artificial neuron (Fig. 1E). As this MOF neuron resembles the modulatory dynamics of biological neurons, sophisticated biomimetic functions were achieved: (i) I&F dynamics (Fig. 1F) and synaptic facilitation-induced spike broadening (Fig. 1G) were achieved by using this MOF neuron; (ii) DA-tunable spiking number (Fig. 1H) and width (Fig. 1I) were realized; (iii) DA-mediated spikes were further implemented to finely control peripheral equipment.

## RESULTS AND DISCUSSION

### DA-mediated synaptic plasticity

**Figure 2.**
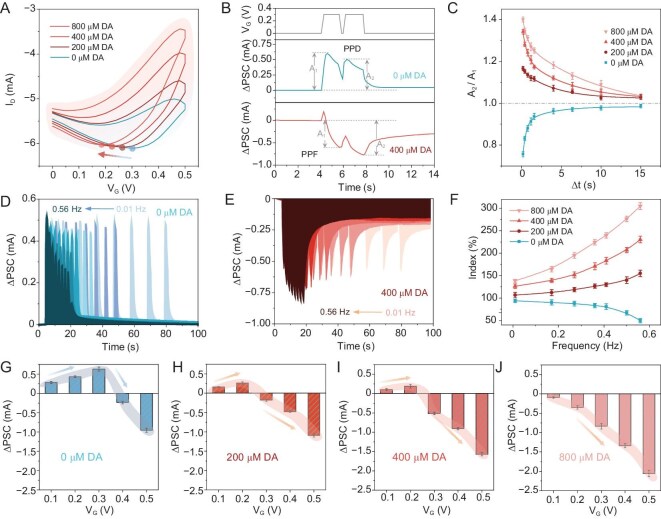
DA-mediated plasticity. (A) Hysteresis characteristic of the device in the presence of different DA concentrations (0, 200, 400, 800 μM). (B) EPSC/PPD and IPSC/PPF behaviors at Δ*t* of 0.4 s in the presence of 0 and 400 μM DA, respectively. (C) Corresponding PPF/PPD indices and the fitting curves. The SRDP in the presence of (D) 0 μM and (E) 400 μM DA under eight presynaptic pulses of 0.3 V and the frequency changed from 0.01 to 0.56 Hz. (F) Corresponding SRDP indices. (G–J) SIDP under the modulation of *V*_G_ ranging from 0.1 to 0.5 V in the presence of different DA concentrations (0, 200, 400, 800 μM).

Synaptic plasticity represents the fundamental function of biological nervous systems in the formation of learning, memory and behavioral planning that are closely regulated by the neurotransmitter [40]. Flexible facilitation/depression of the synaptic weight has proven pivotal in both biological systems and neuromorphic devices [41,42]. To investigate such properties, the effect of DA on the hysteresis characteristics of the MOF transistor was first studied (Fig. 2A). As DA increased from 0 to 800 μM, the channel responses (*I*_D_) and the loop areas were gradually increased due to the enhanced faradaic currents from DA oxidation (Figs S10 and S11) [43,44], indicating the DA-gated plasticity. Notably, the enhanced gating effect was accompanied by a leftward shift in the turning point of the hysteresis curve, with the initial value of ∼0.3 V at 0 μM DA. In such a scenario, paired-pulse facilitation (PPF) and paired-pulse depression (PPD)—a typical form of short-term plasticity that refers to the facilitation/inhibition responses of the second spikes compared with those of the first spikes—could be chemically achieved by using DA modulation. Specifically, two presynaptic pulses at certain time intervals (Δ*t*) were applied to record the excitatory/inhibitory postsynaptic currents (EPSC/IPSC) (Fig. 2B). As shown, at a voltage of 0.3 V and Δ*t* of 0.4 s, the EPSC with PPD behavior was observed at 0 μM DA, i.e. the second postsynaptic currents (PSC) (*A*_2_), was smaller than the previous one (*A*_1_). However, in the presence of 400 μM DA, the IPSC with a PPF behavior was observed, i.e. *A*_2_ was larger than *A*_1_. Such a phenomenon can be attributed to the leftward shift of the turning point induced by the DA. The corresponding index (*A*_2_/*A*_1_) was derived to reveal the degree of facilitation/depression (Fig. 2C). Incidentally, the DA-mediated transition between PPF and PPD was further confirmed (Fig. S12). As the Δ*t* increased from 0.2 to 15 s, both the PPF and PPD indices exhibited the biexponential behavior as fitted by the curves. As the DA increased from 0 to 200 μM, the index transitioned from PPD to PPF. When the DA increased further from 200 to 800 μM, the PPF gradually increased, indicating the DA-enhanced facilitation. These results demonstrated the chemically tunable short-term facilitation and depression characteristics of the MOF device.

In a biological nervous system, spike-rate-dependent plasticity (SRDP) plays a key role in information processing due to the dynamic filtering functions [12,45]. This characteristic could be emulated by applying eight presynaptic pulses of 0.3 V at different frequencies from 0.01 to 0.56 Hz. As shown, at 0 μM DA, the device exhibited EPSC under the pulse trains, which was significantly decreased at high frequency, while the changes were small at low frequency (Fig. 2D), indicating the low-pass filtering function. In the presence of 400 μM DA, it exhibited IPSC under the pulse trains, which was significantly increased at high frequency, while the changes were small at low frequency (Fig. 2E), indicating the high-pass filtering function. Additionally, the SRDP indices (*A*_8_/*A*_1_) at different levels of DA (0, 200, 400, 800 μM) were investigated (Fig. 2F). As the DA increased from 0 to 200 μM, the SRDP index trend was changed from depression to facilitation, indicating that the function transitioned from low-pass to high-pass filtering. When the DA increased from 200 to 800 μM, the SRDP index trend exhibited enhanced facilitation. Such behaviors indicated DA-mediated dynamic filtering functions that were similar to the biological ones that SRDP was chemically dependent on the neurotransmitter [46]. In addition, DA-mediated spike-intensity-dependent plasticity (SIDP) was also simulated. As shown, it exhibited EPSC in *V*_G_ values ranging from 0.1 to 0.3 V and IPSC in *V*_G_ values ranging from 0.4 to 0.5 V at 0 μM DA (Fig. 2G). In the presence of 200 μM DA, the PSC at *V*_G_ of 0.3 V was changed from EPSC to IPSC (Fig. 2H). As the DA increased to 800 μM, the PSC at *V*_G_ of 0.2 and 0.1 V was further changed from EPSC to IPSC (Fig. 2I and J).

### Logic gate computing

**Figure 3.**
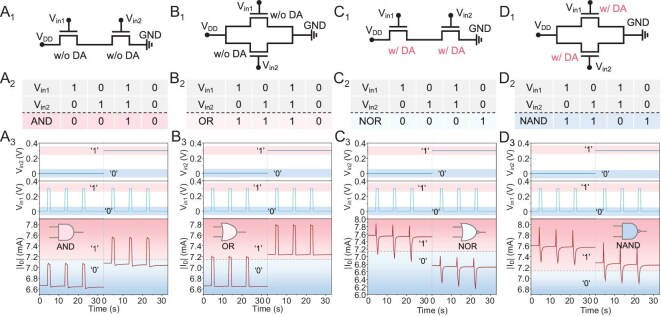
Design of logic gates. (A_1_) Schematic of two MOF devices in series in the absence of DA. (A_2_) Truth table of AND logic gate. (A_3_) Corresponding *I*_D_ of AND logic gate with voltage inputs. (B_1_) Schematic of two MOF devices in parallel in the absence of DA. (B_2_) Truth table of OR logic gate. (B_3_) Corresponding *I*_D_ of OR logic gate with voltage inputs. (C_1_) Schematic of two MOF devices in series in the presence of 800 μM DA. (C_2_) Truth table of NOR logic gate. (C_3_) Corresponding *I*_D_ of NOR logic gate with voltage inputs. (D_1_) Schematic of two MOF devices in parallel in the presence of 800 μM DA. (D_2_) Truth table of NAND logic gate. (D_3_) Corresponding *I*_D_ of NAND logic gate with voltage inputs. *V*_DD_ was set to −0.8 V in parallel and to −0.2 V in series. The input with a voltage of 0.3 V representing ‘1’ and a voltage of 0 V representing ‘0’.

Information processing in biological systems relies on signal integration that is similar to Boolean logic computing [21,47,48]. Inspired by this, standard logic gates based on two MOF devices in series or in parallel, including AND, OR, NOR and NAND, were chemically operated in the absence and presence of DA, where two voltage inputs constituted *V*_in1_ and *V*_in2_, with a voltage of 0.3-V input representing ‘1’ and a voltage of 0-V input representing ‘0’. Firstly, the AND logic gate was achieved by connecting two MOF devices in series in the absence of DA (Fig. 3A_1_) and the corresponding truth table was listed (Fig. 3A_2_). Specifically, the *I*_D_ for inputs of ‘10’, ‘01’ and ‘00’ were all below the set threshold, resulting in an output of ‘0’. By contrast, only an input of ‘11’ could lead to an output of ‘1’ (Fig. 3A_3_). The OR logic gate was achieved by connecting two MOF devices in parallel in the absence of DA (Fig. 3B_1_). The *I*_D_ for an input of ‘00’ was below the set threshold, while inputs of ‘10’, ‘01’ and ‘11’ could all generate a large *I*_D_ above the threshold, resulting in an output of ‘1’ (Fig. 3B_2_ and B_3_). The NOR logic gate was achieved by connecting two MOF devices in series in the presence of 800 μM DA (Fig. 3C_1_). In this case, only an input of ‘00’ could generate a large *I*_D_ above the threshold, resulting in an output of ‘1’, and inputs of ‘10’, ‘01’ and ‘11’ led to an output of ‘0’ (Fig. 3C_2_ and C_3_). The NAND logic gate was achieved by connecting two MOF devices in parallel in the presence of 800 μM DA (Fig. 3D_1_). Inputs of ‘10’, ‘01’ and ‘00’ could all lead to an output of ‘1’ and only an input of ‘11’ could lead to an output of ‘0’ (Fig. 3D_2_ and D_3_). Together, these results clearly indicated the possibility of the design of various logic gates with the assistance of rational chemical mediation. Incidentally, the corresponding transfer curves of the MOF devices in the absence and presence of DA mediation were recorded (Fig. S13) and the inverter function was also realized (Fig. S14).

### DA-mediated MOF neuron

**Figure 4.**
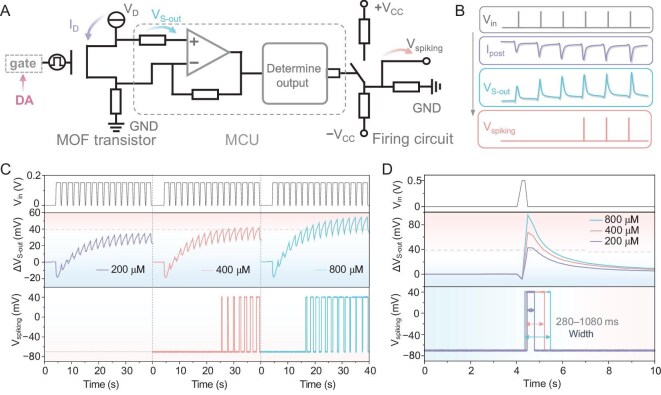
DA-mediated MOF neuron. (A) Circuit diagram of the artificial neuron consists of a MOF transistor, an MCU and an external circuit. (B) Corresponding signal transmission process. (C) DA-mediated I&F and spike-broadening model in the presence of different DA concentrations (200, 400, 800 μM). (D) DA-tunable spike width in the presence of different DA concentrations (200, 400, 800 μM). The three resistors in the firing circuit were all equal to 100 kΩ.

Neurons process and transmit information via spikes that follow neurotransmitter-mediated I&F [5]. The diverse firing behaviors of the spikes, including number and width, are collectively influenced by voltage, ions and neurotransmitters. Inspired by this, we further constructed a MOF neuron by integrating the MOF transistor with an MCU via an external circuit (Fig. 4A). Specifically, the DA can cause an enhanced gating effect and thus changes in the assigned potential (*V*_S-out_) of the MOF transistor in the circuit with the assistance of a resistor; the *V*_S-out_ could be monitored and then evaluated by using the MCU to determine the output. As the amplitude of the biological spikes generally varied from approximately −70 to 40 mV [5,14], we tuned the firing circuit to the same amplitude by distributing two external voltages (+*V*_CC_ of 260 mV and −*V*_CC_ of −140 mV) to three resistors. The corresponding signal transmission process was displayed in detail (Fig. 4B). Specifically, the voltage pulse train was applied, which was subsequently converted into *I*_D_ and then *V*_S-out_. Note that, because of the aforementioned synaptic plasticity, *V*_S-out_ exhibited signal integration that could exceed the set threshold, leading to the opening of the pathway of +*V*_CC_ and thus disturbing the original potential distribution towards generating the spikes.

Based on such a MOF neuron, synaptic facilitation-induced spike broadening [5,49–51] and dynamic I&F were realized under DA mediation (Fig. 4C). As shown, in the presence of 200 μM DA, the integrated signal was below the set threshold under the 16 pulses train of 0.15 V and the MOF neuron maintained the resting potential of –70 mV. As the DA increased to 400 μM, the integrated signal gradually exceeded the threshold under the same pulses train, leading to the generation of seven spikes, with a clear spike-broadening behavior due to the enhanced facilitation. As the DA further increased to 800 μM, the integrated signal rapidly exceeded the threshold, leading to the generation of 11 spikes with similar spike-broadening behavior. In biological neurons, the spike width varies from neuron to neuron and from region to region, encoding different information [5]. In the present MOF neuron, its spike width could also be directly tuned by DA concentrations (Fig. 4D). As shown, in the presence of 200 μM DA, the MOF neuron could generate a spike with a width of 280 ms under a pulse input of 0.5 V, while the presence of 400 and 800 μM DA could extend the width to 790 and 1080 ms, respectively. These results demonstrated the DA-mediated spike behaviors of this MOF neuron. Incidentally, the read energy of a spike with a width of 280 ms was calculated as ∼12 nJ, derived from the product of its width, voltage amplitude of 110 mV and current of 400 nA at a spike potential of 40 mV.

**Figure 5.**
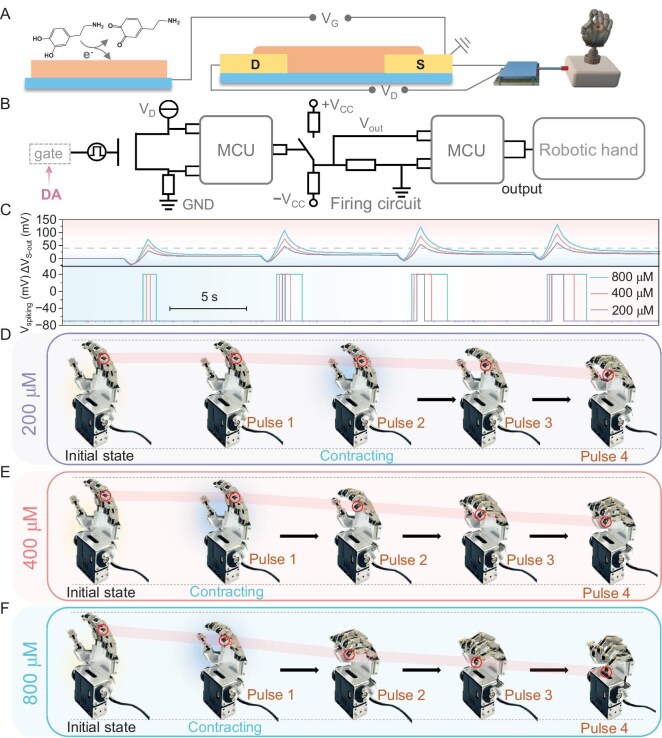
DA-mediated MOF nerve. (A) Schematic of the artificial nerve that contains a MOF neuron and a robotic hand. (B) Diagram of simplified circuit of the MOF nerve. (C) DA-tunable spikes from the MOF neuron for fine control of the robotic hand under four pulses of 0.3-V input. Corresponding contraction processes of the robotic hand in the presence of (D) 200, (E) 400 and (F) 800 μM DA.

An artificial nerve [52] was then constructed by using the MOF neuron to control a robotic hand (Fig. 5A). Under DA mediation, on-demand spikes of the MOF neuron could be produced and sent into another MCU to manage the robotic arm (Fig. 5B). As the DA increased from 200 to 800 μM, the facilitation of the MOF neuron was increased under four pulses of 0.3-V input, leading to an increase in the spike widths (Fig. 5C) for fine control of the robotic arm. Specifically, in the presence of 200 μM DA, the robotic arm began to slightly contract at a pulse number of two. As the pulse number increased to four, the robotic arm continuously contracted to approximately two-thirds of its initial state (Fig. 5D). When the DA was increased to 400 μM, the robotic arm began to slightly contract at the first pulse and gradually contracted to half its initial state when the pulse number increased to four due to the increased spike width (Fig. 5E). As the DA increased to 800 μM, the robotic arm exhibited significant contraction at the first pulse, while it completed the contraction at a pulse number of four (Fig. 5F). Incidentally, the corresponding processes of contraction were recorded (Video S1).

## CONCLUSION

In summary, we report a proof-of-concept study of a MOF neuron with DA perception in fluids. The solution-processed MOF transistor exhibited DA-mediated synaptic plasticity including PPF/PPD and spike-dependent plasticity, which further realized the basic logic gates of AND, OR, NOR and NAND via the combination of configuration design and DA presence. Based on integrating the MOF transistor with an MCU, a MOF neuron was achieved that was capable of simulating I&F dynamics, synaptic facilitation-induced spike broadening, DA-tunable spiking number and spiking width in aqueous environments. DA-mediated spikes in this MOF neuron were further implemented to finely control peripheral equipment, which could potentially be explored for future neuromorphic biosensing and artificial neural networks. This is a report of the first MOF neuron with chemical intelligence in liquids, demonstrating the potential of the large family of MOFs for artificial neuron applications.

## METHODS

### Materials and reagents

Nickel chloride hexahydrate (NiCl_2_·6H_2_O) and aqueous ammonia (NH_3_·H_2_O, 14 mol L^−1^) were purchased from Sinopharm Chemical Reagent Co., Ltd (Shanghai, China). 2,3,6,7,10,11-Hexaaminotriphenylene hexahydrochloride (HATP·6HCl) was obtained from Bide Pharmatech Ltd (Shanghai, China). Dopamine (DA) was purchased from Sigma-Aldrich (Shanghai, China). All aqueous solutions were prepared with ultrapure water (18.2 MΩ·cm) and filtered with 0.22-μm filter membranes.

### Fabrication of the MOF transistor

To pattern the glass substrate, 10-nm-thick chromium (Cr) and 100-nm-thick gold (Au) layers were sequentially deposited by using magnetron sputtering (Kurt J. Lesker Company) on clean polyethylene glycol terephthalate (PET) substrates through a patterned shadow mask with width 6.0 mm and length 0.2 mm. Subsequently, the MOF membrane was *in situ* deposited onto the PET substrates. Specifically, 6.6 mg (0.028 mmol) of NiCl_2_·6H_2_O was fully dissolved in 5 mL of H_2_O as Solution A; 0.3 mL of NH_3_·H_2_O was then added to a solution of 10 mg (0.019 mmol) of HATP·6HCl in 5 mL of H_2_O as Solution B. Subsequently, Solutions A and B were mixed and stirred continuously for 5 min. The patterned PET substrates were placed on the mixture solution and floated on the liquid surface, with the patterned side facing the solution. Afterwards, the reaction mixture was transferred to an oven and kept at 65°C for 30 min, followed by washing of the fabricated MOF film with ultrapure water three times. Last, except for the MOF film at the channel area, redundant MOF film was scraped. Finally, the fabricated MOF transistor was annealed at 100°C for 2 h in a glovebox with an argon atmosphere and allowed to cool naturally.

### Characterization

The electrical measurements were performed on a Keithley 4200 semiconductor parameter analyser by using 0.1 M KCl as the electrolyte. Scanning electron microscope images were obtained from a Hitachi S4800 microscope. Powder X-ray diffraction patterns were obtained by using Bruker D8 Advance equipment. Ultraviolet–visible absorption spectra were obtained by using a Shimadzu UV-3600 Ultraviolet–visible–near-infrared spectrophotometer. Atomic force microscopic images were recorded by using a Bruker Icon atomic force microscope (Bruker Corporation, America). The X-ray photoelectron spectroscopy spectra were obtained on an ESCALAB 250Xi spectrometer (Thermo-VG Scientic Co. USA).

## Supplementary Material

nwaf213_Supplemental_Files
