# Supplementary material for: A metal–organic framework neuron
Source: Natl Sci Rev. 2025 May 23;12(7):nwaf213. doi: 10.1093/nsr/nwaf213 (PMC12278819; doi:10.1093/nsr/nwaf213)
Supplement: nwaf213_Supplemental_Files [file nwaf213_supplemental_files.zip › Supplementary data.pdf]

Supporting Information for:

A Metal-Organic Framework Neuron

*Zheng Li, Miao-Hua Chen, Qing-Qing Wu, Cheng Yuan, Jing-Juan Xu, Hong-Yuan Chen and Wei-Wei Zhao**

State Key Laboratory of Analytical Chemistry for Life Science, School of Chemistry and Chemical Engineering, Nanjing University, Nanjing 210023, China

E-mail: zww@nju.edu.cn

*To whom correspondence should be addressed.

Contents

Figure S1. Synthesis of $Ni_3(HITP)_2$.

Figure S2. The morphology of as-prepared MOF.

Figure S3. XRD patterns of the MOF.

Figure S4. XPS patterns of the MOF.

Figure S5. The UV/vis spectra of $Ni_3(HITP)_2$ film.

Figure S6. Electrical characteristic and stability of the MOF transistor.

Figure S7. The transfer characteristic curves of the MOF transistor measured under different KCl concentrations.

Figure S8. The transfer characteristic curves of the MOF transistor measured under different channel voltage.

Figure S9. The transfer characteristic curves of the MOF transistor measured under different concentrations of DA.

Figure S10. The corresponding redox reaction of the MOF with DA.

Figure S11. Cyclic voltammograms of the MOF film.

Figure S12. The DA-mediated transition between PPF and PPD.

Figure S13. The transfer characteristic curves of the MOF transistors used in the logic gates.

Figure S14. Inverter function based on the MOF devices.

Table S1. Some artificial neuron devices.

As shown, the HATP 6HCl was treated with an aqueous solution of $NiCl_2$ $6H_2O$ under air, followed by the addition of NH_4OH under constant stirring. The mixture was then heated to 65 ℃, generating the dark blue-violet film of $Ni_3(HITP)_2$ (Fig. S1).

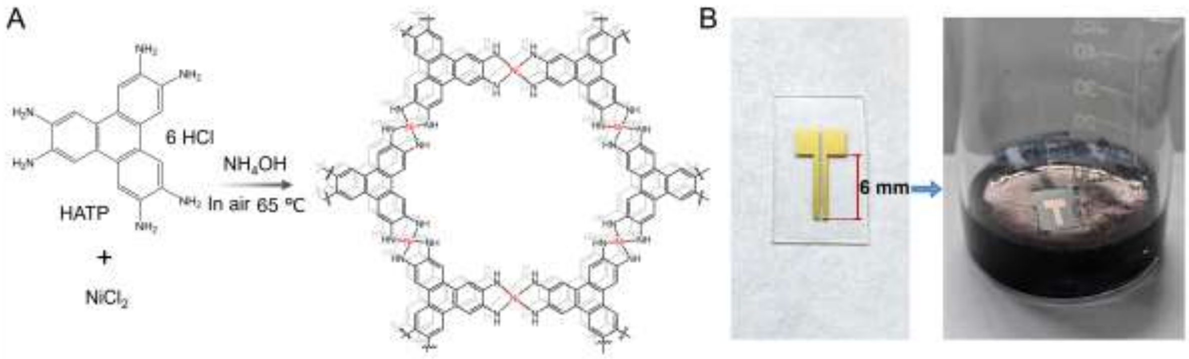

Figure S1. Synthesis of $Ni_3(HITP)_2$. A) The structure of $Ni_3(HITP)_2$. B) Photograph of the corresponding preparation process.

As shown, the MOF composed of numerous nanoparticles was revealed by a scanning electron microscope (SEM) (Fig. S2A). The thick of the MOF membrane on PET was revealed by an atomic force microscope (AFM) (Fig. S2B), and the thick of the MOF membrane was ca. 120 nm (Fig. S2C).

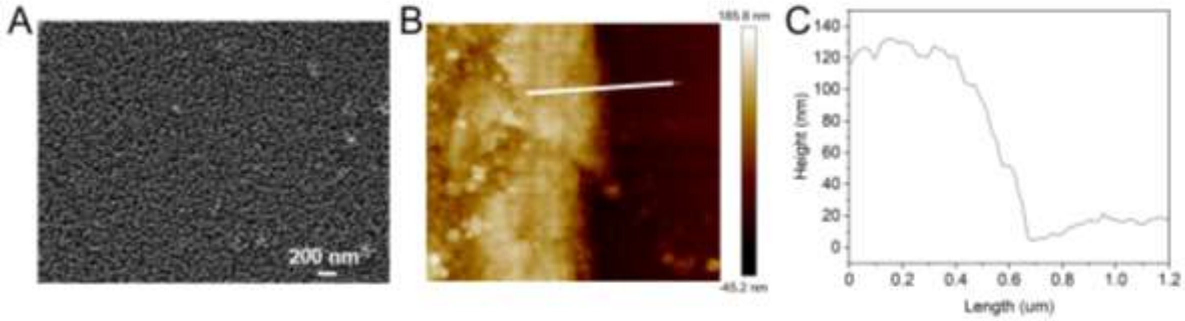

Figure S2. The morphology of as-prepared MOF. (A) SEM image of the MOF. (B) AFM image of the MOF membrane on PET. (C) The corresponding thick of the MOF membrane.

X-ray diffraction (XRD) spectrum of the $Ni_3(HITP)_2$ MOF was displayed, as shown, the MOF featured characteristic peaks at 4.7 °, 9.5 °, 12.6 °, 16.5 ° and 27.3 ° (Fig. S3), which matched well with the previous report [1].

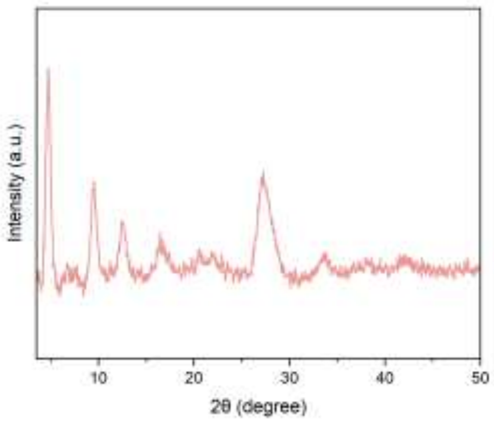

Figure S3. XRD patterns of the MOF.

As shown, the X-ray photoelectron spectroscopy (XPS) results (Fig. S4A and S4B) revealed that the Ni $2p_{3/2}$ peak on the $Ni_3(HITP)_2$ membrane was located at 855.3 eV and had very weak broad satellite peak, which was the same as that of pure $Ni_3(HITP)_2$ crystallite in previous report [2].

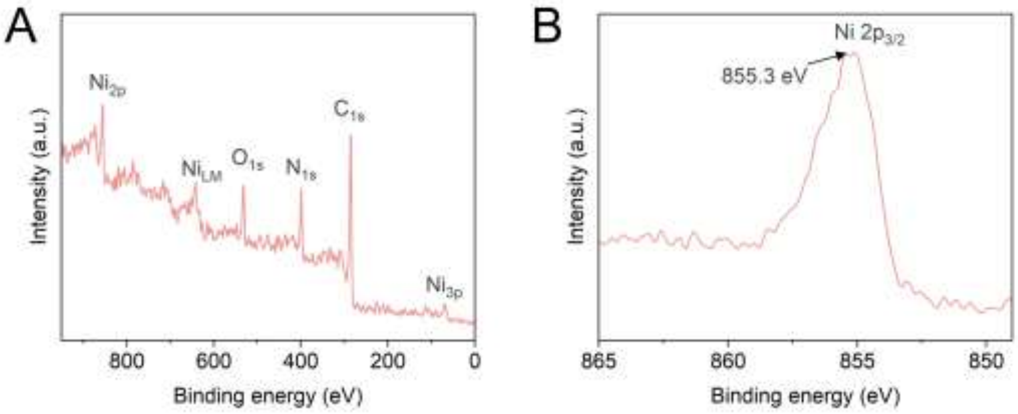

Figure S4. XPS patterns of the MOF.

The UV-vis spectra indicated the formation of the $Ni_3(HITP)_2$ on the substrate. As shown, the absorbance exhibited two characteristic absorbance peaks at ca. 320 nm and 652 nm corresponding to π–π* transition and ligand-to-metal charge-transfer transition, respectively (Fig. S5) [3].

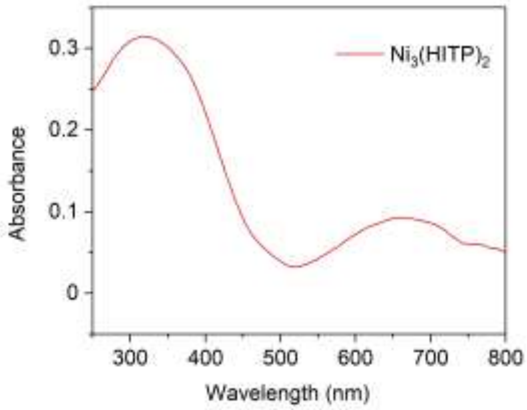

Figure S5. The UV/vis spectra of $Ni_3(HITP)_2$ film.

The channel characteristics were first investigated using an Ag/AgCl electrode, and the corresponding transfer curve was recorded (Fig. S6A). In order to verify the stability of the as-fabricated MOF transistor, we measured the transfer curves for 480 min. As shown, the transfer curves measured in different time were quite consistent (Fig. S6B).

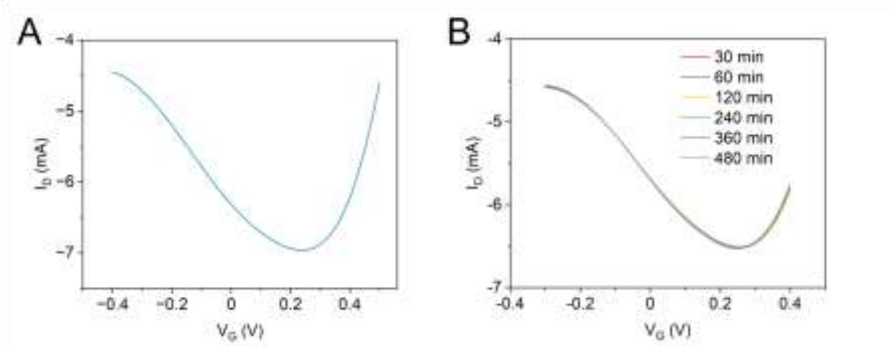

Figure S6. A) The transfer characteristics gated by an Ag/AgCl. B) Electrical stability of the MOF transistor.

As shown, the transfer characteristic curves of the MOF transistor measured under the different KCl concentrations from 10 to 100 mM, the transfer characteristic curves exhibited a leftward trend (Fig. S7), indicating the enhanced gating effect.

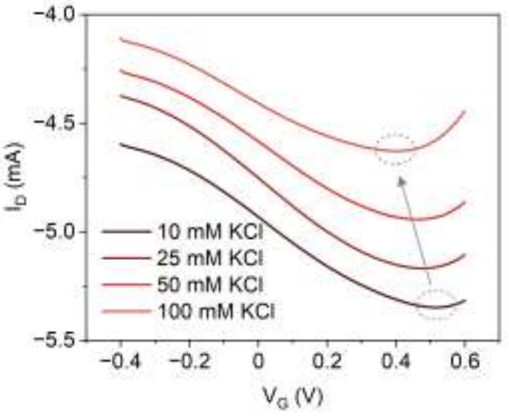

Figure S7. The transfer characteristic curves of the MOF transistor measured under different KCl concentrations.

Similarly, the channel voltage had an effect on the gating effect of the transistor. As shown, as the channel voltage increased from −0.1 to −0.7 V, the transfer characteristic curves exhibited a leftward trend, and the turning point was tuned to 0 V when the channel voltage was -0.7 V (Fig. S8).

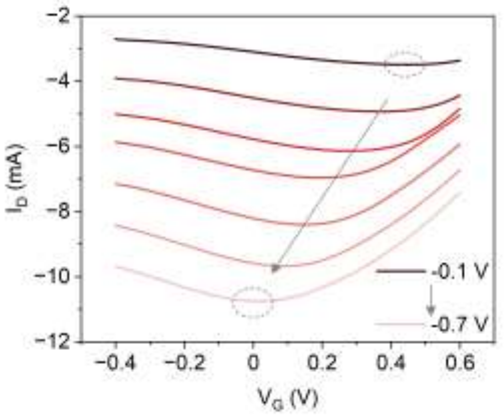

Figure S8. The transfer characteristic curves of the MOF transistor measured under different channel voltage.

The transfer characteristic curves exhibited a gradual leftward trend as the DA increased from 0 to 800 μM, indicating the enhanced gating effect (Fig. S9).

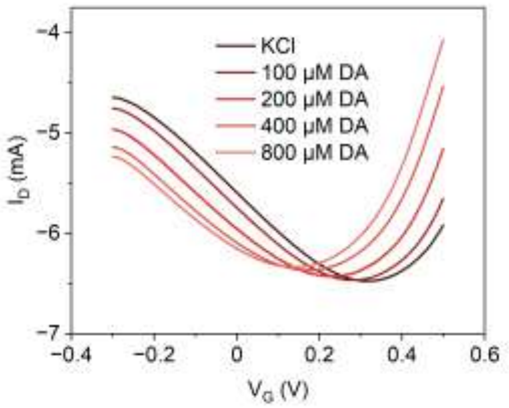

Figure S9. The transfer characteristic curves of the MOF transistor measured under different concentrations of DA.

The MOFs with radical *o*-semiquinonate ligands have been known to show a rich electrochemical activity, undergoing both oxidation and reduction on the radical *o*-diiminobenzosemiquinonate ligands, which was available for electrochemical oxidation of DA (Fig. S10) [4, 5].

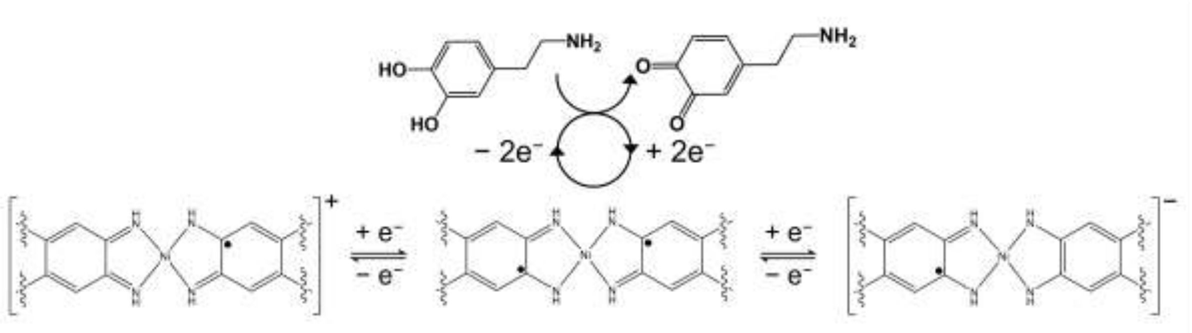

Figure S10. The corresponding redox reaction of the MOF with DA.

Cyclic voltammetry tests were employed to confirm the electrochemical reaction (Fig. S11). As shown, in the potential range 0 to 0.5 V, the oxidation currents increased gradually as the increase of DA from 0 to 800 μM, demonstrating the DA oxidation.

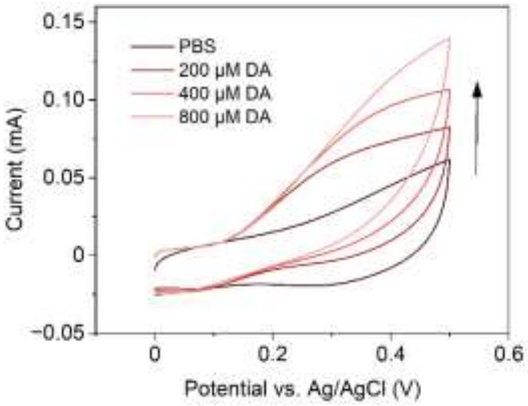

Figure S11. Cyclic voltammograms of the MOF film in PBS before and after the additions of DA with different concentrations. The scan rate is 50 mV/s.

The DA-mediated transition between PPF and PPD was further confirmed (Fig. S12). As shown, at the voltage of 0.3 V and Δt of 1.2 s, the index exhibited a PPD behavior at 0 μM DA and a PPF behavior at 200 μM DA. As the DA increased from 200 to 400 μM, the index increased gradually, demonstrating the reliability of the DA-mediated transition from PPD to PPF.

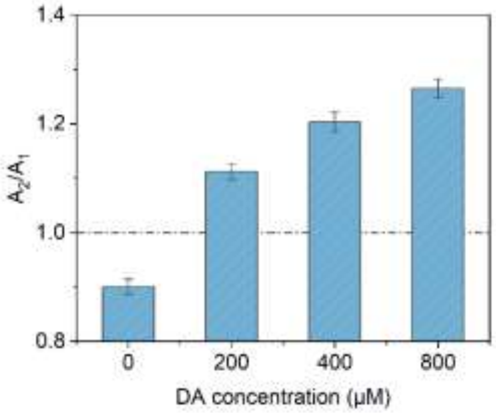

Figure S12. The PPF/PPD index at the Δt of 1.2 s in the presence of different DA concentrations (0, 200, 400, 800 μM).

The transfer characteristic curves of the MOF transistors used in the logic gates were recorded (Fig. S13). As shown, in the shaded area, the transfer characteristic curves exhibited an upward trend and a downward trend in the absence and presence of 800 μM DA, respectively.

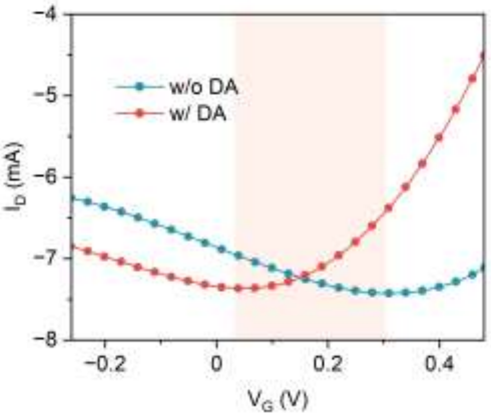

Figure S13. The transfer characteristic curves of the MOF transistors with/without 800 μM DA.

Inverter function was also realized in the MOF device (Fig. S14). As shown, the proposed inverter was composed of the pristine MOF device and the device with 800 μM DA modulation. The output voltage (V_{out}) decreased with the increase of the input voltage (V_{in}), demonstrating the inverter effect.

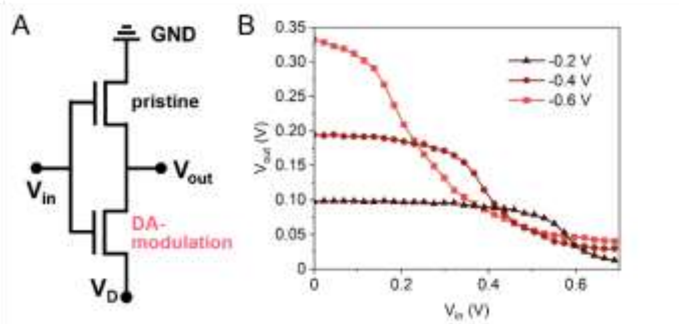

Figure S14. The inverter consists of a pristine MOF device and a DA-modulated device. A) Schematic of the inverter. B) Voltage transfer characteristics at different V_D.

Table S1. Some artificial neuron devices

Neuromorphic devices	Channel materials	Neurotransmitter perception	Neurotransmitter tunable neuron spikes	Neurotransmitter controlled actuator	Ref.
Memristor	Ag nanoparticles–silk fibroin	DA	No	Yes, depend on DA concentrations	[6]
Organic transistor	BBL	acetylcholine	Yes, frequency	No	[7]
Organic transistor	BBL and P($g_4$2T-T)	No	No	No	[8]
Field-effect transistor	MoS_2 and CNT	No	No	No	[9]
MOF transistor	MOF	DA	Yes, width and number	Yes, depend on DA concentrations	This work

References

1. Sheberla D, Sun L, Blood-Forsythe MA *et al.* High electrical conductivity in Ni_3(2,3,6,7,10,11-hexaiminotriphenylene)$_2$, a semiconducting metal–organic graphene analogue. *J Am Chem Soc* 2014; **136**: 8859-8862.
2. Wu G, Huang J, Zang Y *et al.* Porous field-effect transistors based on a semiconductive metal–organic framework. *J Am Chem Soc* 2017; **139**: 1360-1363.
3. Song X, Wang X, Li Y *et al.* 2D semiconducting metal–organic framework thin films for organic spin valves. *Angew Chem, Int Ed* 2020; **59**: 1118-1123.
4. Noro S-i, Chang H-C, Takenobu T *et al.* Metal−organic thin-film transistor (MOTFT) based on a bis(o-diiminobenzosemiquinonate) nickel(ii) complex. *J Am Chem Soc* 2005; **127**: 10012-10013.
5. Ko M, Mendecki L, Eagleton AM *et al.* Employing conductive metal–organic frameworks for voltammetric detection of neurochemicals. *J Am Chem Soc* 2020; **142**: 11717-11733.
6. Wang T, Wang M, Wang J *et al.* A chemically mediated artificial neuron. *Nat Electron* 2022; **5**: 586-595.
7. Harikesh PC, Yang C-Y, Wu H-Y *et al.* Ion-tunable antiambipolarity in mixed ion–electron conducting polymers enables biorealistic organic electrochemical neurons. *Nat Mater* 2023; **22**: 242-248.
8. Harikesh PC, Yang CY, Tu D *et al.* Organic electrochemical neurons and synapses with ion mediated spiking. *Nat Commun* 2022; **13**: 901.
9. Beck ME, Shylendra A, Sangwan VK *et al.* Spiking neurons from tunable gaussian heterojunction transistors. *Nat Commun* 2020; **11**: 1565.
